# Supplementary material for: VPS18 recruits VPS41 to the human HOPS complex via a RING–RING interaction
Source: Biochem J. 2017 Oct 23;474(21):3615–26. doi: 10.1042/BCJ20170588 (PMC5651818; doi:10.1042/BCJ20170588)
Supplement: Supplementary Material [file BCJ-474-3615-s1.pdf]

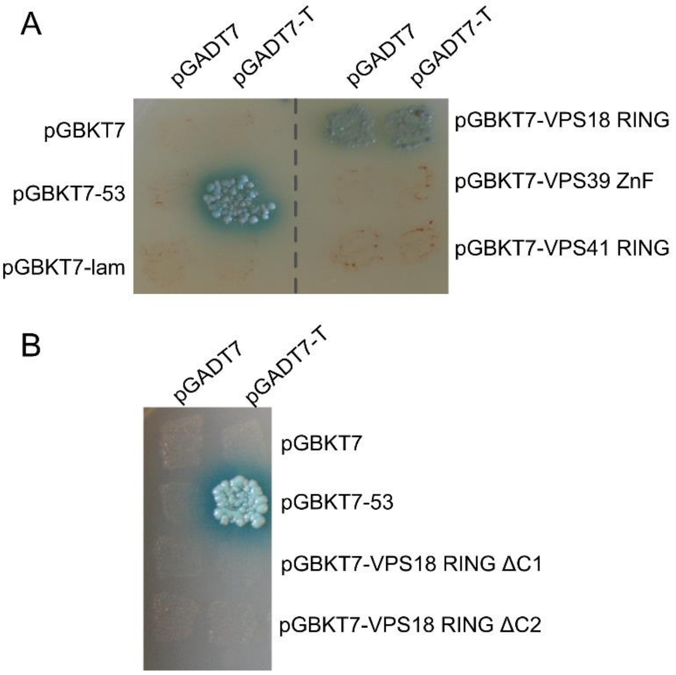

Supplementary figure 1: Auto-activation testing of HOPS RING domains for yeast two-hybrid experiments. (A) Yeast strain Y2H Gold was transformed with a Gal4 DNA binding domain fused to VPS18 and VPS41 RING domains (pGBKT7-VPS18 RING and pGBKT7-VPS41 RING), to the VPS39 zinc finger domain (pGBKT7-VPS39 ZnF), to a positive control (human p53; pGBKT7-53), to a negative control (human lamin C; pGBKT7-lam), or in isolation (pGBKT7). Strain Y187 was transformed with the Gal4 activation domain in isolation (pGADT7) or fused to the SV40 large T antigen (pGADT7-T). Yeast were mated and then grown on -Leu -Trp -Ade -His nutritional selection media supplemented with X-α-Gal and aureobasidin A, growth of blue colonies indicating a positive interaction. (B) Two truncated VPS18 RING domain constructs where the putative C-terminal helix had been removed (VPS18 RING ΔC1 and ΔC2, spanning residues 842–957 and 842–961, respectively) were tested for auto-activation as described in (A).

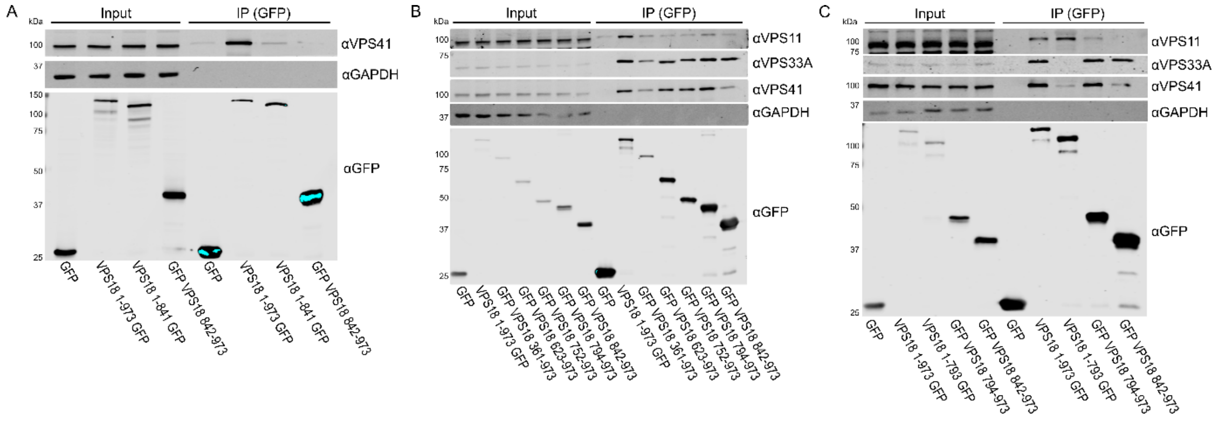

Supplementary figure 2: Co-immunoprecipitations of various VPS18 truncation constructs expressed in HEK293T cells. (A) The VPS18 (1-841) construct retains some interaction with endogenous VPS41. (B) A series of N-terminal truncations of VPS18 shows that extended RING domain constructs are more efficient at co-immunoprecipitating endogenous VPS41 than the RING domain alone (842-973). (C) VPS18 (1-793) co-immunoprecipitates endogenous VPS11 with an efficiency equal to full length VPS18 (1-973).

Supplementary data: Proteins identified as putative HOPS RING domain interaction partners by yeast two-hybrid.

VPS18 RING yeast two-hybrid hits	Tested in vitro by GST pulldown
Homo sapiens ATPase, Na+/K+ transporting, beta 3 polypeptide	
Homo sapiens ATPase, Na+/K+ transporting, beta 1 polypeptide, mRNA	
Homo sapiens protein arginine methyltransferase 5 (PRMT5)	
Homo sapiens eukaryotic translation initiation factor 1B (EIF1B)	
Homo sapiens voltage-dependent anion channel 2 (VDAC2), transcript variant 3	
protein tyrosine phosphatase, receptor type, M	
Homo sapiens procollagen-lysine, 2-oxoglutarate 5-dioxygenase 1 (PLOD1)	
Homo sapiens cytochrome b reductase 1 (CYBRD1), transcript variant 3	
Homo sapiens transforming growth factor, beta receptor III (TGFBR3)	
Homo sapiens fibrillin 1 (FBN1)	
Homo sapiens enolase 1, (alpha) (ENO1), transcript variant 2	
Homo sapiens electron-transfer-flavoprotein, alpha polypeptide	
Homo sapiens spastic paraplegia 21 (autosomal recessive, Mast syndrome)	
Homo sapiens ash2 (absent, small, or homeotic)-like (Drosophila) (ASH2L), transcript variant X2	
Homo sapiens ubiquitin specific peptidase 1 (USP1), transcript variant 1	
Homo sapiens ubiquitin specific peptidase 31 (USP31), transcript variant X1	
Homo sapiens leucine rich repeat containing 37, member A3	
proline rich 16	
Homo sapiens peptidylprolyl isomerase A (cyclophilin A)	
solute carrier family 30 (zinc transporter), member 9 Target Range : 42031601->42032363	
myomesin 2	
Homo sapiens unc-13 homolog B (C. elegans) (UNC13B), transcript variant X6	
FLJ23158 fis, clone LNG09623; Uncharacterized protein	
GUF1 GTPase homolog (S. cerevisiae)	
dickkopf WNT signaling pathway inhibitor 3	
DnaJ (Hsp40) homolog, subfamily B, member 1 OR trans-2,3-enoyl-CoA reductase	
Homo sapiens nibrin (NBN), transcript variant X3	
enhancer of yellow 2 homolog (Drosophila)	
family with sequence similarity 35, member A	
Homo sapiens calbindin 1, 28kDa (CALB1), transcript variant X1	
Homo sapiens tissue-type breast unknown protein	
tRNA methyltransferase 11 homolog (S. cerevisiae)	
pantothenate kinase 3	
Homo sapiens DEP domain containing MTOR-interacting protein (DEPTOR), transcript variant 2	
Homo sapiens AU RNA binding protein/enoyl-Coenzyme A hydratase	
Homo sapiens ER lipid raft associated 2 (ERLIN2), transcript variant X1	
tetratricopeptide repeat domain 29	
Homo sapiens nuclear receptor subfamily 4, group A, member 3 (NR4A3)	
Homo sapiens genomic DNA, chromosome 11 clone:RP11-113K21	
Homo sapiens zinc finger with KRAB and SCAN domains 4 (ZKSCAN4), transcript variant X7	
Homo sapiens chromosome 19 clone CTD-2105E13	
Homo sapiens clone DNA64905 olfactomedin-like (UNQ663)	
Homo sapiens phosphoprotein enriched in astrocytes 15	
Human DNA sequence from clone RP11-25C17 on chromosome 6	
Homo sapiens tropomodulin 3 (ubiquitous) (TMOD3)	
Homo sapiens spermatogenesis associated 18 (SPATA18)	
Homo sapiens murine retrovirus integration site 1 homolog (MRVI1), transcript variant X10	
Homo sapiens nudix (nucleoside diphosphate linked moiety X)-type motif 2 (NUDT2), transcript variant 3	
Homo sapiens ring finger protein 139 (RNF139)	

Homo sapiens adaptor-related protein complex 1, gamma 1 subunit (AP1G1), transcript variant X6
Homo sapiens N(alpha)-acetyltransferase 38, NatC auxiliary subunit (NAA38)
Homo sapiens isolate ny135 mitochondrion, complete genome
Human DNA sequence from clone RP11-298E9 on chromosome 10
Homo sapiens mediator complex subunit 13 (MED13), transcript variant X1
Homo sapiens transcription factor IIB
Homo sapiens ribosomal protein S20
DNM1 pseudogene 47
Homo sapiens membrane associated guanylate kinase, WW and PDZ domain containing 1 (MAGI1)
Homo sapiens RAN binding protein 6
Homo sapiens dynactin 6 (DCTN6)
Homo sapiens DEAD (Asp-Glu-Ala-Asp) box polypeptide 1
Homo sapiens UDP-glucose ceramide glucosyltransferase (UGCG), transcript variant X1
Homo sapiens protein translation factor sui1 homolog
Homo sapiens translational factor eIF-1
Human DNA sequence from clone RP11-393K12 on chromosome 10
Homo sapiens fibulin 5

VPS41 RING yeast two-hybrid hits	Tested in vitro by GST pulldown
proliferating cell nuclear antigen	
thymine-DNA glycosylase	
phosphoglucomutase 1 (PGM1), transcript variant 3	Yes, no interaction
chromosome 2 open reading frame 42	Yes, no interaction
latent transforming growth factor beta binding protein 2	
HKR1, GLI-Kruppel zinc finger family member	
cytokine receptor-like factor 3 (CRLF3), transcript variant 2	Yes, no interaction
ATPase, Na+/K+ transporting, beta 3 polypeptide	Yes, no interaction
Niemann-Pick disease, type C2 (NPC2)	Yes, no interaction
ATPase, Na+/K+ transporting, beta 1 polypeptide	Yes, no interaction
oxysterol binding protein-like 1A (OSBPL1A)	Yes, no interaction
adenosylhomocysteinase (AHCY), transcript variant 1	
sterile alpha motif domain containing 9-like	
vacuolar protein sorting 13 homolog C (S. cerevisiae) (VPS13C)	
Homo sapiens ovostatin homolog 2-like	
pumilio homolog 2 (Drosophila)	
BAC clone RP11-640B6 from 4	
cell division cycle associated 7-like	
sperm associated antigen 17	
WNT1 inducible signaling pathway protein 1 (WISP1)	
dynactin 6 (DCTN6)	
FRAS1 related extracellular matrix 1 (FREM1)	
LIM and calponin homology domains 1	
tetratricopeptide repeat domain 3 pseudogene 1	
Homo sapiens interleukin 33	
Homo sapiens chromosome 9 CRA_219000002707971	

VPS39 ZnF yeast two-hybrid hits	Tested in vitro by GST pulldown
Homo sapiens phosphatidylinositol 4-kinase type 2 beta	
Homo sapiens cDNA FLJ45203 fis, clone BRCAN2008701 CDC40	
Homo sapiens lectin, galactoside-binding, soluble, 1 (LGALS1)	Yes, no expression
Homo sapiens keratin 222 (KRT222)	
Homo sapiens arrestin domain containing 5 (ARRDC5)	Yes, no interaction
unc-51-like kinase 4 (C. elegans) pseudogene 2	
flavin containing monooxygenase 2 (non-functional)	
Homo sapiens phosphoglucomutase 1 (PGM1)	Yes, no interaction
Homo sapiens protein phosphatase 2 (formerly 2A), catalytic subunit, beta isoform	
ubiquitin specific peptidase 15/family with sequence similarity 19 (chemokine (C-C motif)-like), member A2	
Homo sapiens splicing factor, arginine/serine-rich 5	
Homo sapiens ATPase, Na+/K+ transporting, beta 1 polypeptide	Yes, no interaction
Homo sapiens cDNA: FLJ22650 fis, clone HSI07344	
Homo sapiens carboxypeptidase E (CPE)	
Homo sapiens protein associated with topoisomerase II homolog 1 (yeast) (PATL1)	
Homo sapiens small nuclear ribonucleoprotein polypeptide G pseudogene 2	
Homo sapiens cDNA FLJ75025 complete cds, highly similar to Homo sapiens peptidylprolyl isomerase A (cyclophilin A) (PPIA)	
Homo sapiens proliferating cell nuclear antigen	
Homo sapiens cDNA FLJ75832 complete cds, highly similar to Homo sapiens TRAF-interacting protein with a forkhead-associated domain (TIFA)	
Homo sapiens dynein, cytoplasmic 1, intermediate chain 2 (DYNC1I2), transcript variant 5 1546-2154	Yes, no interaction
